# Gitelman and Bartter Syndrome in a Patient With Morbid Obesity: A Case Report and Literature Review

**DOI:** 10.1002/ccr3.71903

**Published:** 2026-02-04

**Authors:** Solmaz Hasani, Alireza Rezapanah, Tooraj Zandbaf, Mohammad Javad Ghamari, Narges Mesbah

**Affiliations:** ^1^ Endocrine Research Center, School of Medicine Mashhad University of Medical Sciences Mashhad Iran; ^2^ Surgical Oncology Research Center, Faculty of Medicine Mashhad University of Medical Sciences Mashhad Iran; ^3^ Department of General Surgery, MMS. C. Islamic Azad University Mashhad Iran; ^4^ Innovative Medical Research Center, MMS. C. Islamic Azad University Mashhad Iran

**Keywords:** bariatric and metabolic surgery, Bartter syndrome, case report, Gitelman syndrome

## Abstract

We present a case study of a 34‐year‐old man with morbid obesity and a suspected Bartter–Gitelman spectrum tubulopathy (without genetic confirmation), weighing 135 kg, and with a BMI of 42.5 kg/m^2^, who was referred to the metabolic and bariatric surgery department due to morbid obesity to address abnormal electrolyte levels. The clinical presentation suggested renal tubular salt wasting. Post‐sleeve gastrectomy, the patient had sustained and prolonged normalization in his electrolytes without the use of any medications. This provides evidence of a possible relationship between metabolic surgery and renal tubular function. This case supports recognizing a suspected tubulopathy in candidates for metabolic surgery.

## Key Clinical Message

This case underscores the importance of recognizing suspected Bartter–Gitelman spectrum tubulopathy in morbidly obese surgical candidates presenting with unexplained electrolyte disturbances.

## 1 Introduction

Bartter syndrome (BS) and Gitelman syndrome (GS) are salt‐losing renal tubular disorders affecting the excretion and reabsorption of sodium, potassium, and chloride [1, 2]. The original understanding of BS and GS posited them as genetically and clinically distinct conditions. However, current data suggest they are better conceptualized as a spectrum of a single disease. This spectrum is a consequence of mutations in diverse genes that code for proteins responsible for chloride reabsorption at varying locations throughout the nephron [3, 4, 5].

BS represents a group of genetically heterogeneous disorders with five subtypes reported, and GS is caused by variants in genes encoding ion transporters or channels, most commonly causing loss of function [4, 5, 6, 7, 8, 9]. GS in approximately 80%–95% of patients is associated with mutations in SLC12A3 encoding thiazide‐sensitive Na‐Cl cotransporter (NCCT) [8, 10]. The metabolic disturbances of BS and GS include hypokalemia, hypochloremic metabolic alkalosis, and normal or reduced blood pressure despite hyperreninemia and hyperaldosteronism [11]. Urinary calcium excretion can be a differentiating feature, with hypocalciuria being typical in GS and hypercalciuria in BS. Though previously considered a specific characteristic of GS, hypomagnesemia has also been documented in some patients with BS [12, 13]. Juxtaglomerular apparatus hyperplasia is typically observed in BS [11]. Patients being evaluated for Metabolic and Bariatric Surgery (MBS) frequently present with a myriad of metabolic disturbances that can overlap with the manifestations of rare genetic conditions, such as GS or BS. These disorders, characterized by an inherited defect in renal electrolyte handling, present with symptoms such as hypokalemia and metabolic alkalosis, which can be easily misattributed to other conditions often seen in the preoperative work‐up of MBS candidates [14, 15].

MBS is fundamentally predicated on the understanding that severe obesity is a disease entity with numerous detrimental health consequences that can be ameliorated or reversed through significant weight loss. This approach is typically considered for individuals whose nonsurgical weight loss interventions have been unsuccessful [16]. This case report will detail an instance of a patient who was being assessed for MBS, and he was suspected to have Bartter–Gitelman spectrum tubulopathy.

## 2 Case History

### 2.1 Case Presentation

A 34‐year‐old man, with a suspected Bartter–Gitelman spectrum tubulopathy (clinical diagnosis without genetic confirmation), weighing 135 kg and with a BMI of 42.5 kg/m^2^, was referred to the MBS department due to morbid obesity to address abnormal electrolyte levels. The patient's medication history includes spironolactone 25 mg taken three times a day, calcium carbonate 500 mg taken eight times a day, magnesium 250 mg taken five times a day, and propranolol. Laboratory tests before surgery revealed the following abnormalities: hypokalemia (3 mg/dL), hypomagnesemia (1.7 mg/dL), and hypocalcemia (7.6 mg/dL). After consulting with a local MBS commission, the patient was deemed a suitable candidate for surgery. Standard preoperative diagnostic procedures and consultations were performed, leading to the recommendation for Sleeve Gastrectomy.

### 2.2 Surgical Findings

After preoperative evaluations, the patient was prepared for surgery. A laparoscopic sleeve gastrectomy was performed under general anesthesia and with the standard method.

### 2.3 Postoperative Course

After the surgery, the patient exhibited Trousseau and Chvostek's signs indicating hypocalcemia. He received 1 g of calcium gluconate, and the issue was resolved. The day after surgery, methylene blue and UGI tests with gastrografin were done, and both were normal. The patient consumed a clear fluid diet during their hospitalization, the patient was discharged in stable condition and without any problems. His lab test on the third day showed calcium was 10 mg/dL, potassium 2/9 mg/dL, and magnesium 1.6 mg/dL. The case received detailed dietary guidelines, medication instructions, and personalized exercise recommendations to facilitate his recovery and long‐term weight management. He returned to the clinic 10 days later with no complaints, and his vital signs and abdominal exam were normal.

Three months after the surgery, the patient had a normal electrolyte status without needing specific treatment.

## 3 Discussion and Conclusion

The metabolic disturbances associated with BS and GS include hypokalemia, hypochloremic metabolic alkalosis, and normal or reduced blood pressure despite hyperreninemia and hyperaldosteronism [11]. Patients with these biochemical abnormalities may report a range of clinical symptoms. The most frequently documented complaints include muscle cramps, weakness, fatigue, and tetany. Furthermore, thirst and salt cravings are common due to renal salt wasting, leading to hypovolemia; these symptoms are not specific [17, 18]. Methods for the diagnosis of GS and BS are: clinical suspicion is necessary to perform a diagnostic test, but clinical criteria do not offer a certain diagnosis. The diuretic test is simple to perform and inexpensive; however, it can pose life‐threatening risks to the patient. The study of microvesicles is a noninvasive test that is not part of standard clinical procedures and requires specialized personnel to perform. Genetic diagnosis is the most reliable diagnostic technique complicated by large rearrangements [19]. Our patient has hypokalemia, hypomagnesemia, and hypocalcemia, and he was asymptomatic at presentation.

GS, once considered a variant of BS, is now recognized as an independent entity following the successful identification of the gene responsible for its manifestation [20]. Despite these similarities, they diverge in their molecular origins and clinical presentations. BS represents a group of genetically heterogeneous disorders with five subtypes reported, and GS is caused by variants in genes encoding ion transporters or channels, most commonly causing loss of function (Table 1) [4, 5, 6, 7, 8, 9]. GS is often discovered incidentally during routine blood testing when hypokalemia is detected. A defining feature of GS is hypocalciuria, which may be associated with ectopic calcification. Conversely, increased urinary magnesium excretion may contribute to hypomagnesemia. The salt‐wasting pattern seen in BS tends to be more pronounced than in GS, with diagnosis typically occurring during infancy or early childhood. BS is often characterized by hypercalciuria, potentially leading to renal stone formation or calcification. Urine and serum magnesium levels in BS patients are generally normal or low [21]. However, it is important to note that not all BS patients manifest with low serum magnesium levels. A preliminary distinction can be attempted using the urinary calcium/creatinine ratio. For practical considerations, a chloride clearance test employing hydrochlorothiazide and furosemide can differentiate these conditions, and this convenient and cost‐effective test is useful in various healthcare settings [18]. Pharmacological treatments for Bartter and Gitelman syndromes are supplemental electrolyte drugs, NSAIDs, potassium‐sparing diuretics, and inhibitors of RAAS. Growth hormone is designed to prevent growth abnormalities in children. It is important to note that all current treatments are supportive and cannot address the primary issues later in life [19].

**TABLE 1:** Gitelman syndrome and Bartter syndrome classification.

Disorder	Gene	Protein
BS type 1	SLC12A1	NMCC2
BS type 2	KCNJ1	K1.1
BS type 3	CLCNKB	CLC‐Kb
BS type 4a	BSND	Barttin
BS type 4b	CLCNKA and CLCNKB	CLC‐Ka—CLC‐Kb
BS type 5	MAGEDD2	MAGE‐D2 antigen
GS	SLC12A3	NCCT

The guidelines, informed by established standards of preoperative and perioperative management for surgical patients, also offer tailored recommendations pertinent to the specific needs of this patient across various aspects of care. These aspects include the preoperative assessment process, the minimum acceptable preoperative potassium and magnesium levels, and the necessary intra‐ and post‐procedural monitoring. To develop evidence‐based recommendations, the guideline group conducted extensive investigations into the interplay of anesthesia, hypokalemia, and hypomagnesemia. The insights gained were then applied to the specific context of salt‐wasting alkalosis. Crucially, the group determined that a patient‐specific risk assessment is paramount. This assessment should take into account factors such as the nature of the surgery, concomitant medications—specifically digoxin, insulin, and β‐adrenoceptor agonists—and any existing cardiovascular risk factors. The presence of preexisting cardiac dysrhythmias was identified as a critical factor, supported by findings from a prospective study involving 10,000 surgical patients. This study revealed that a history of dysrhythmia, rather than chronic hypokalemia (2.6–3.4 mmol/L), predisposed individuals to intraoperative dysrhythmias. The guideline group also recommends that monitoring during anesthesia and recovery should largely adhere to national guidelines established for the general population. Furthermore, systems must be in place to facilitate prompt assessment and correction of any electrolyte imbalances, with continuous ECG monitoring available throughout the recovery period. While day‐case surgery may be appropriate for stable patients undergoing minor procedures, for more extensive surgical interventions, such as abdominal hysterectomy, endoscopic prostatic resection, lumbar discectomy, and thyroidectomy, immediate postoperative electrolyte measurements, followed by subsequent testing as clinically indicated, are recommended. Finally, postoperative high‐dependency care should be available, including facilities for serial intra‐arterial potassium sampling, for higher risk patients. These patients include those with unstable potassium concentrations, those for whom paralytic ileus is anticipated, or those at risk of exacerbated hypokalemia due to fluid losses from vomiting or other causes [22, 23]. It is noticeable Fujino reported a case of rectal Cancer in a Patient with Bartter Syndrome. After surgery to resect the tumor, his hypokalemia was easier to control [24].

### 3.1 Proposed Mechanism

The observed normalization of electrolytes following the operation may relate to improved renal tubular function, reduced renin‐angiotensin‐aldosterone system activation, and improved insulin sensitivity after the loss of weight. However, these mechanisms are potential, hypothetical, and should be tested in future studies.

This case is particularly unusual because there is both morbid obesity and suspected salt‐wasting tubulopathy, a combination that is rarely reported. Unlike previous cases that primarily focused on electrolyte abnormalities or surgical complications, this report highlights the postoperative normalization of electrolytes following bariatric surgery. This observation raises the possibility of improved renal tubular function or altered hormonal regulation as potential contributors. We conducted a brief literature review of case reports regarding these syndromes to emphasize their various aspects (Table 2).

**TABLE 2:** Brief literature review.

	Age	Symptoms	Laboratory findings	Comorbidity	Interventions
Our study	34	—	Hypokalemia, hypomagnesemia, and hypocalcemia	Morbid obesity	Surgery
Huang et al. [25]	36	Numbness in the hands and feet, as nausea, vomiting, abdominal distension, chest tightness, or palpitations	Erythrocytosis	Diabetes mellitus	Potassium chloride solution, spironolactone, regimen comprising insulin glargine, gliclazide sustained‐release tablets, acarbose, and sitagliptin
Fujino et al. [24]	51	Anemia	Hypokalemia, hypomagnesemia	Rectal cancer	Mics remained stable perioperatively. Her serum potassium level stabilized after surgery
ALSaleh et al. [26]	39	Recurrent attacks of acute pancreatitis	Normal	Heart failure, diabetes mellitus, hypothyroidism, latent tuberculosis, and pulmonary embolism	Exploratory laparotomy
Nagmoti et al. [27]	22	Occipital headache, paraesthesia of right upper and lower limbs	Hypokalemia	Arnold–Chiari malformation	Surgery, correct metabolic alkalosis
Jiang et al. [28]	25	Bilateral lower limb paralysis, numbness, and weakness	Severe hypokalemia, hyperkaliuria, metabolic alkalosis, and high renin and aldosterone levels	Episode of muscle paralysis due to a severe hypokalemia	Spironolactone and potassium chloride

### 3.2 Limitation

This case lacks genetic or functional diagnostic confirmation, such as diuretic testing or gene sequencing. Therefore, the diagnosis remains clinical and we would prefer to frame it as a suspected tubulopathy in the Bartter–Gitelman spectrum. This diagnostic ambiguity about the diagnosis, and future research is required to confirm such cases using genetic testing.

### 3.3 Conclusion

Evidence suggests that bariatric surgery may be beyond weight reduction, potentially influencing renal electrolyte handling in patients with suspected Bartter–Gitelman spectrum tubulopathy. However, due to the absence of genetic confirmation and mechanistic insights, these findings should be interpreted cautiously. Further studies with genetic and functional analyses are required to better understand this association.

## Author Contributions

**Solmaz Hasani:** investigation, writing – original draft. **Alireza Rezapanah:** data curation, writing – original draft. **Tooraj Zandbaf:** data curation, investigation, writing – review and editing. **Mohammad Javad Ghamari:** investigation, writing – original draft. **Narges Mesbah:** investigation, writing – review and editing.

## Funding

The authors have nothing to report.

## Consent

The patient's written consent was obtained for the publication of this case report.

## Conflicts of Interest

The authors declare no conflicts of interest.

## Data Availability

The data supporting this research are available from the authors on reasonable request.
